# Evidence for dysregulation of axonal growth and guidance in the etiology of ASD

**DOI:** 10.3389/fnhum.2013.00671

**Published:** 2013-10-22

**Authors:** Kathryn McFadden, Nancy J. Minshew

**Affiliations:** ^1^Department of Neurobiology, University of PittsburghPittsburgh, PA, USA; ^2^Department of Psychiatry and Neurology, University of Pittsburgh Medical SchoolPittsburgh, PA, USA

**Keywords:** autism spectrum disorders, connectivity, neuritic outgrowth, axonal guidance, subplate

## Abstract

Current theories concerning the cause of autism spectrum disorders (ASDs) have converged on the concept of abnormal development of brain connectivity. This concept is supported by accumulating evidence from functional imaging, diffusion tensor imaging, and high definition fiber tracking studies which suggest altered microstructure in the axonal tracts connecting cortical areas may underly many of the cognitive manifestations of ASD. Additionally, large-scale genomic studies implicate numerous gene candidates known or suspected to mediate neuritic outgrowth and axonal guidance in fetal and perinatal life. Neuropathological observations in postmortem ASD brain samples further support this model and include subtle disturbances of cortical lamination and subcortical axonal morphology. Of note is the relatively common finding of poor differentiation of the gray–white junction associated with an excess superficial white matter or “interstitial” neurons (INs). INs are thought to be remnants of the fetal subplate, a transient structure which plays a key role in the guidance and morphogenesis of thalamocortical and cortico-cortical connections and the organization of cortical columnar architecture. While not discounting the importance of synaptic dysfunction in the etiology of ASD, this paper will briefly review the cortical abnormalities and genetic evidence supporting a model of dysregulated axonal growth and guidance as key developmental processes underlying the clinical manifestations of ASD.

## INTRODUCTION

Autism spectrum disorders (ASDs) are characterized by deficits across apparently disparate domains; language, social reciprocity, sensory integration, and repetitive/restricted behavior patterns among others. Within each domain, however, functioning is often markedly uneven, and the coexistence of significant impairments with areas of normal or even enhanced performance is a long recognized paradox. Cognitive-neurologic testing (Minshew et al., 1997, Minshew et al., 2002; Williams et al., 2006) has indicated the common denominator across domains is a normal or enhanced ability to perform perceptual and simple information processing tasks coupled with significant deficits in the ability to perform tasks requiring complex information processing, even in high-functioning, high-IQ subjects with ASD. Rather than implicating dysfunction in a particular brain area/structure, this cognitive profile is most consistent with altered functioning of the distributed cortical neural network, i.e., how and how well cortical functional areas, particularly association areas, communicate with each other and their subcortical targets (Minshew and Payton, 1988). This model of aberrant connectivity in ASD is now widely accepted, although the details vary (e.g., Belmonte et al., 2004; Geschwind and Levitt, 2007).

Functional magnetic resonance imaging (fMRI), which allows for examination at the neural systems level, has been key to demonstrating the above impairments and modeling altered connectivity in ASD. Numerous fMRI studies have reported decreased synchronization of critical cortical areas during the performance of complex tasks (or at rest) in subjects with ASD relative to age and IQ-matched controls. This appears to be particularly marked in tasks requiring high functional connectivity between frontal association (e.g., anterior cingulate and prefrontal cortices), and more posterior cortical regions (reviewed in Schipul et al., 2011) such as complex sentence comprehension (Just et al., 2004), social inference (Just et al., 2007), or inhibition (Kana et al., 2007).

**FIGURE 1:**
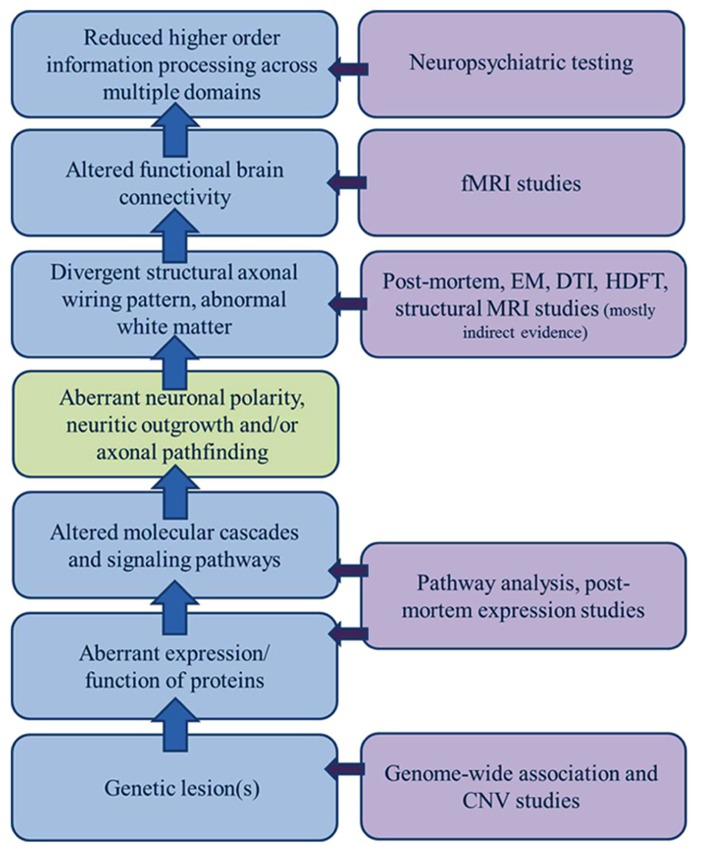
**Sources of evidence for a model of dysregulated axonal development in ASD.** A broad gap exists between the clinical manifestations of ASD and growing list of genetic candidates; a gap only incompletely bridged by a general model of altered connectivity. A significant component of dysregulated axonal development (polarity, outgrowth, and guidance/targeting should be considered in constructing a model of ASD etiology that encompasses all of the clinical, functional, structural, and genetic observations.

Altered anatomic connectivity is the most likely substrate for reduced functional connectivity (**Figure 1**), although the exact basis of the relationship is not established and much debated. At the physical level, neural circuitry is comprised of neurons, their processes (axons and dendrites), and their synapses on neighboring or distant neurons. Wiring the brain, therefore, requires the coordinated interactions of numerous molecular cascades and environmental exposures during development so that neurons proliferate, migrate to the appropriate locations, extend axons with a high degree of spatial and temporal fidelity, and establish synaptic connections with appropriate target neurons. Clearly, one or more of these developmental processes does not unfold in the typical fashion in ASD. In recent years, much attention has been paid to altered synaptic function as the central event explaining altered brain connectivity in ASD. However, the view of ASD as predominately an intrinsic synaptopathy is unsatisfying in view of the cerebral white matter alterations documented in many subjects and outlined below.

## ALTERED BRAIN GROWTH TRAJECTORIES: EVIDENCE FROM STRUCTURAL MRI STUDIES

Structural MRI, morphometric, and neuropathologic studies provide ample evidence of altered neocortical growth and organization in ASD. Studies examining head circumference and brain volume in individuals with ASD have demonstrated altered brain growth trajectories across the lifespan. While not significantly different from controls at birth, up to 70% of infants later diagnosed with ASD exhibit abnormally accelerated brain growth in the first year of life (Courchesne et al., 2003). Approximately 20–25% of infants in this subset meet formal criteria for macrocephaly in the first year. Brain volume ascertained by MRI is significantly larger in 90% of infants with ASD by 2–4 years of life as well (Courchesne et al., 2003). Many studies note a marked rostral–caudal gradient in these altered growth trajectories (reviewed in Lainhart, 2006). At the time of maximal brain growth in very early childhood, cerebral gray matter and white matter are both increased (by approximately 20 and 40%, respectively). The frontal cortical gray and white matter show the most enlargement followed by the temporal and parietal lobes. The occipital gray and white matter and parietal gray matter tend not to vary significantly from normal (Carper et al., 2002). Within the frontal lobes, the gray matter areas most affected are the dorsolateral and mesial prefrontal cortex. Similarly, the white matter most involved appears to be the radiate compartment and U-fibers immediately underlying these cortical areas which represent intrahemispheric, cortico-cortical connections originating from cortical layers II and III (Herbert et al., 2003). The corpus callosum, conversely, is often reduced in autism (e.g., Hardan et al., 2000; Keary et al., 2009). Therefore, increased brain size in toddlers with ASD appears to be largely driven by enlargement of the white matter compartment underlying the frontal and temporal cortices (Carper et al., 2002; Herbert et al., 2004; Carper and Courchesne, 2005).

Following this initial acceleration, growth rates decline significantly causing an apparent normalization of brain volume by adolescence and early adulthood (Courchesne et al., 2001, Courchesne et al., 2004; Waiter et al., 2005). This relative decrease is most marked in the white matter in that children with autism experience only a 10% increase in cerebral white matter between the ages of 3 and 12 years (Courchesne et al., 2001). Gray matter volumes remain elevated into adulthood as does mean head circumference. Rates of macrocephaly, although lower, remain increased overall. This pattern stands in stark contrast to the age related cerebral white matter increase (60%) and gray matter decrease observed in typically developing individuals between the ages of 4 and 22 years (Tau and Peterson, 2010) which are generally thought to be a function of the concurrent processes of synaptic/collateral pruning and myelination. It must be noted that the above pattern does not hold true for all individuals with ASD. Many show typical rates of head and brain growth and a small subset even meet criteria for microencephaly, although this is more common in the setting of syndromic ASD (see Activating Mutations in the mTOR Pathway are Associated with Syndromic ASD for discussion of this concept).

## ALTERED CORTICAL MICROSTRUCTURE: EVIDENCE FROM POSTMORTEM STUDIES

A number of studies have reported gross and microscopic changes that may relate to increased gray matter and alterations in relative compartmental volumes. One recent report of seven autistic children with increased brain size (Courchesne et al., 2011) involved a 67% increase in the numbers of neurons in the prefrontal cortex relative to age-matched controls. Neurogenesis, the birth and early proliferation of neurons, is largely a prenatal process. At birth, cortical neurons are typically small, so that an appreciable excess might not translate into a significant change in head size. However, in the first years of life, the typical dramatic increase in cytoplasmic volumes (both of the cell body and axons/dendrites) occurring in more than the usual complement of frontal neurons, could account for abnormally accelerated brain growth in the first years of life. It would also explain why maturational synaptic/neuritic pruning may not register as an appreciable loss of cerebral gray matter in these individuals.

Related to this is the finding of subtle microstructural abnormalities in cortical architecture, even in the absence of more obvious dysgenic lesions. Although most of these analyzes are not rigorously stereologic, the impression is of increased numbers of narrow minicolumns containing increased densities of neurons (Casanova et al., 2002, 2006; Buxhoeveden et al., 2006) in the frontal cortex of ASD brains. This trend appears to be most pronounced in the frontal lobe, particularly the dorsolateral prefrontal cortex, and is not seen in more posterior regions such as the visual cortex. Minicolumns are the vertical cell columns created by sequential waves of migrating neurons traveling along radial glial fibers during early corticogenesis. Increased numbers of such arrays may reflect excess early divisions of radial glial cells immediately prior to the onset of neurogenesis and migration. Furthermore, the distribution of minicolumn abnormalities correlates with patterns of accelerated growth and excess neurons in the early postnatal period.

More than half of all postmortem investigations have uncovered additional features of cortical dysgenesis, presumably caused by abnormal neurogenesis, neuronal migration or maturation, in ASD brains. Bailey et al. (1998) observed significant and widespread cortical dysgenic lesions in four of six subjects with ASD. Similarly, in a recent large-scale study, Wegiel et al. (2010) reported a wide variety of dysgenic lesions and heterotopias in multiple cortical regions in 12 of 13 subjects with ASD. These included excess subependymal neurons, subcortical and periventricular heterotopias, and additional minor disruptions of cytoarchitecture. In the seminal postmortem studies by Kemper and Bauman (Bauman and Kemper, 1985, 1998; Kemper and Bauman, 1993, 2002), the only consistent abnormality in the cerebral cortex was relatively small neuronal cell size and increased cell packing in the anterior cingulate cortex. Simms et al. (2009) also found decreased cell size and decreased cell packing in different sub-regions of the anterior cingulate. Van Kooten et al. (2008) conducted a stereologic study on the fusiform gyrus, involved in face processing, and found significantly lower neuronal densities within layer III and lower total neuron numbers in layers III, V, and VI, as well as smaller average cell volumes of neurons in layers V and VI. Hutsler and Zhang (2010) found increased dendritic spine densities in the temporal lobes of individuals with ASD and intellectual disability relative to age-matched controls. Most of the reported cortical microstructural finding in ASD are very subtle. It must be noted that even the non-subtle dysgenic lesions reported in the literature are not specific to ASD and are more often found in non-ASD individuals both with and without seizures or other neurologic symptoms. Conversely, the vast majority of ASD brains show relatively normal cortical cytoarchitecture.

## ALTERED CORTICAL WHITE MATTER: EVIDENCE FROM DTI AND POSTMORTEM STUDIES

It is very possible that an increase in absolute numbers and densities of neurons in the frontal cortex could have an adverse effect on anterior–posterior connectivity. The mismatch created by relatively too few afferent and/or too many efferent axonal terminals attempting to form circuits could potentially be disruptive. But converging lines of evidence also point to microstructural differences in white matter. Much of this evidence is indirect and relatively non-specific, but white matter is notoriously hard to study. While MRI-based studies allow the direct examination of the general course and volume of major white matter tracts, they have lacked the necessary resolution to directly examine even large axon fascicles or to trace these entering the cortex. Histologic techniques, conversely, have permitted the direct examination of axons and their myelin coverings, but are too laborious and time consuming to feasibly trace connections over large distances in the brain. Fortunately, recent advances in high definition fiber tracking (HDFT) promise to soon permit the best (or the best compromise) of both worlds. HDFT is a novel tractography method using high-angular-resolution diffusion imaging and diffusion spectrum imaging (DSI) techniques in order to track white matter fibers from cortical origins, through complex fiber crossings, to cortical and subcortical targets with at least millimeter resolution (a few hundred axons; Verstynen et al., 2011; Fernandez-Miranda et al., 2012). Initial reports (only in the popular media for now) have demonstrated significant alterations in the morphology (i.e., wiring plan) of a number of major white matter tracts in a few individuals with ASD (e.g., Schneider, 2011, 2012). These highly preliminary findings indicate the physical alterations of ASD in the brain may prove to be quite unsubtle, but until now, almost completely invisible.

Much of the current evidence for altered white matter comes from studies employing diffusion tensor imaging (DTI). DTI measures apparent diffusibility of water molecules as a function of direction over time and is a method to characterize the organization and microstructural properties of white matter. The most common DTI measure is fractional anisotropy (FA) which characterizes the directional variation in the apparent diffusions. White matter, which is arranged in parallel arrays of axonal bundles (fascicles) tends to have a higher FA than gray matter as diffusion of water in neuropil has less directionality (i.e., is more isotropic). A complementary measure, radial diffusivity (RD), describes the tendency of perpendicular water movement and is, therefore, lower in white matter compared to gray matter (reviewed in Travers et al., 2012). Numerous studies have found widespread decreases in FA (and concurrent increases in RD or similar measures) in children (>4 years of age) and adults with ASD (reviewed in Travers et al., 2012). This tendency is most pronounced in the corpus callosum (e.g., Shukla et al., 2010; Jeong et al., 2011), cingulum bundle, and various white matter tracts involving the temporal and frontal lobes (e.g., Jou et al., 2011; Shukla et al., 2011) thereby correlating, generally, with both the fMRI and structural MRI growth trajectory data. These white matter alterations are attributed to reduced tract coherence and/or loss of microstructural integrity, but are not specific to a particular etiology as similar effects could potentially be produced by reduced myelination, increased axonal diameter, alterations of axonal density, or more complex white matter (i.e., turning or crossing fibers, or excess branching) or reduced axonal fasciculation (Travers et al., 2012).

Microscopic tissue studies, although limited by the time necessary to perform, allow a greater degree of resolution and may potentially being able to resolve these differences. In a rare and recent study, Zikopoulos and Barbas (2010) stereologically investigated the fine structure (by light and electron microscopy) of myelinated axons in the white matter below the anterior cingulate cortex, orbitofrontal cortex, and lateral prefrontal cortex in individuals with ASD relative to age-matched controls. They found similar overall axonal density between groups below all prefrontal areas. However, the ASD group had significantly fewer large axons (likely representing the more long-range cortico-cortical connections) in the deep white matter below the anterior cingulate cortex. This was associated with the presence of a significantly greater density of smaller axons (thought to connect more adjacent cortical areas) in the corresponding superficial white matter. There were no discernible differences in neuronal densities or distributions in the overlying gray matter. The white matter below the anterior cingulate cortex also exhibited a significantly higher proportion of branched axons of medium caliber. Axons in the superficial white matter below the orbitofrontal exhibited significantly thinner myelin sheaths when controlled for axon diameter despite similar numbers of oligodendrocytes (Zikopoulos and Barbas, 2010). Azmitia et al. (2010, 2011) also found a significant excess of morphologically abnormal serotonin axons in principle ascending fiber bundles of the medialand lateral forebrain bundles as well as target areas in the temporal cortex, amygdala, and globus pallidus.

## THE POTENTIAL ROLE OF THE FETAL SUBPLATE IN WIRING ALTERATIONS IN ASD

Probably the most pervasive cortical finding in ASD, documented in both neuropathologic and structural MRI studies, is the relatively poor differentiation of the gray–white junction associated with excess superficial white matter or interstitial neurons (INs; Bailey et al., 1998; Casanova et al., 2002; Hutsler et al., 2007; Simms et al., 2009; Wegiel et al., 2010; Avino and Hutsler, 2011). This is noted particularly in the white matter just below the frontal association cortices and superior temporal gyrus (e.g., Avino and Hutsler, 2010). The presence of excess INs in ASD has been long attributed to abnormalities of cortical migration despite the very limited findings of gross laminar alterations within the cortex proper. An alternative theory, however, is that these INs represent excess remnants of the fetal subplate instead of arrested neurons destined for the upper layers of the cortical plate (Avino and Hutsler, 2010). This is an attractive hypothesis as this structure performs numerous developmental functions related to formation of neocortical circuits. Interestingly, this finding is also not specific to autism. Increased numbers of INs, particularly in the dorsolateral prefrontal and temporal cortices of schizophrenic patients, have been reported in five of six studies to date, making it one of the most replicated postmortem finding in this disorder as well (Eastwood and Harrison, 2003; Fung et al., 2011; Yang et al., 2011).

The subplate is a transient cortical compartment which becomes fully established during the second trimester and mostly resolves by the sixth postnatal month in humans. Many subplate neurons are actually born before the appearance of the cortical plate proper, and are the first cortical neurons to mature functionally, differentiating into diverse subpopulations in terms of morphology, molecular markers, and neurotransmitter identity (Kostovic and Rakic, 1990). Throughout fetal development, the subplate serves as the major, albeit transient, postsynaptic target for all classes of cortical afferents, both in terms of location of origin and neurotransmitter system (Kanold and Luhmann, 2010). This function is reflected by subplate-enriched or specific expression of numerous extracellular matrix (Chun and Shatz, 1988) and axon guidance molecules, e.g., cadherins, ephrins, semaphorins, and Rho-GTPases (Oeschger et al., 2012).

The earliest afferents, which enter the subplate and synapse on subplate neurons between 8 and 12 gestational weeks, derive from the brainstem nuclei and basal forebrain (Kostovic et al., 2012). Thalamocortical afferents then arrive in huge numbers beginning approximately 13 gestational weeks. All of the above afferents accumulate and remain within the subplate until approximately 22–24 gestational weeks when they begin to penetrate the cortical plate roughly in the order in which they arrived in the subplate (Kostovic and Judas, 2010). At the same time, significant numbers of cortico-cortical and callosal afferents begin arriving in the subplate where they will wait as well. In the case of thalamocortical connections, the most studied in this context, it is thought that the role of the subplate neurons during the “waiting” period is to act as an intermediary between the thalamic neuron and the cortical target thereby relaying thalamic input to layer IV. The subplate neuron then serves as a pioneer axon to guide the afferent to the target cell. The early thalamocortical synapse is weak, but by co-activating the target neuron, the subplate neuron assists in maturing and strengthening the connection (Kanold and Luhmann, 2010). Subplate neurons likely play the same role for other classes of cortical afferents, but this has not been established.

When an adequately strong final synaptic connections are finally established, it is thought that subplate neurons receive an unknown signal to undergo developmental apoptosis. During the perinatal period of subplate dissolution, afferents representing long associative cortico-cortical pathways are still present in the diminishing subplate (Vasung et al., 2010; Kostovic et al., 2012). Subplate dissolution can be seen to begin earlier in primary motor and sensory cortices and later in association areas (e.g., prefrontal cortex and operculum) and coincides somewhat with the development gyral complexity. The postnatal persistence of the subplate in frontal association areas has been related to the ongoing growth of short-range cortico-cortical connections Finally, the subplate is still present in the early postnatal frontal cortex and contains developing short cortico-cortical pathways (Kostović and Judas, 2007; Kostovic et al., 2012).

Neurons surviving dissolution of the subplate persist into adulthood as INs, dispersed among the cortico-cortical “U-fibers” of the superficial white matter. In humans and other primates they remain quite numerous in frontal and prefrontal areas relative to more caudal regions, e.g., visual cortex and are represented by both excitatory, glutamatergic and inhibitory, GABAergic cells (reviewed in Suárez-Solá et al., 2009). What role they may play in adult brain function is unknown, although it is hypothesized that abnormal axonal connectivity during fetal life may cause, or be reflected by, abnormalities in the numbers and/or distribution of INs that persist into adulthood. The presence of excess INs in ASD could potentially be explained by either abnormal proliferation early in embryonic life or reduced developmental apoptosis in the later fetal/perinatal period (Chun and Shatz, 1989; Avino and Hutsler, 2010). Because the subplate is an early structure, the same frontal overgrowth causing excess radial glia/minicolumns and cortical neurons may also be responsible for (or related to) the production of excess subplate neurons. This could potentially be tested. Conversely, subplate neurons not capable of “hooking up” their dependent cortical afferents to the proper targets, for one reason or another, might not receive or properly process the signal for programed cell death. This would be much more difficult to test.

## GENETIC MODELS OF ASD

A predominately genetic etiology for ASD is well established and supported by twin and family studies. An estimated 10–15% of children evaluated for ASD have a known genetic syndrome (e.g., Fragile X or tuberous sclerosis), and an additional 25% or so are found to have an identifiable chromosomal deletion or duplication (i.e., copy number variation, CNV; Sebat et al., 2007). However, despite the recent use of microarray technology to perform CNV analysis and whole genome expression profiling and association studies on large samples, the genetic structure underlying most idiopathic autism is still poorly known. There is considerable debate concerning this architecture, and arguments may be made for either effects of single, rare risk alleles, or interactions of numerous common low-risk alleles. Although these models are not mutually exclusive, only a few identified genetic lesions are recurrent to any appreciable extent. Therefore, the majority of the dozens of candidate loci (and hundreds of associated genes) currently under investigation are derived from rare Mendelian mutations, CNVs, and genes/chromosomal regions associated with syndromic forms of ASD (Marshall and Scherer, 2012). A number of schemes have been generated to organize this growing list in order to both identify a common, underlying pathophysiology as well as point to new potential candidate genes. Most of these models group candidates according to (1) participation in a common signaling pathway, (2) shared molecular or cellular function, or (3) participation in a common developmental pathway.

### ACTIVATING MUTATIONS IN THE mTOR PATHWAY ARE ASSOCIATED WITH SYNDROMIC ASD

Approximately 10–15% of children being evaluated for ASD are found to have a syndromic form, i.e., an ASD or ASD-like behavioral phenotype occurring in the context of a recognized single gene or chromosomal syndrome and/or associated with one or more dysmorphic features (e.g., fragile X or tuberous sclerosis). Many common syndromic disorders with a significant ASD component are caused by alterations in genes that directly or indirectly participate in the mammalian target of rapamycin (mTOR) signaling pathway, i.e., tuberous sclerosis (TSC1/2), fragile X mental retardation 1 (FMR1), neurofibromatosis type 1 (NF1), PTEN mutation syndrome, and Rett’s syndrome (MECP2; reviewed in Levitt and Campbell, 2009). The mTOR signaling pathway serves to integrate extracellular signals (e.g., growth factors) with downstream intracellular activities. In response to upstream tyrosine kinase signaling, ERK and PI3K activate mTOR which, via further kinase signaling, activates multiple downstream genes responsible for cellular proliferation, growth, survival, fate decision, and motility. PTEN, NF1, and TSC1/2 are all inhibitors of mTOR so that their pathogenic mutations all have the downstream effect of increasing mTOR signaling. MECP2 encodes a protein that regulates the transcription of multiple downstream genes involved either directly in the ERK/PI3K pathway or the upstream MET RTK pathway. Again, the net effect of MECP2 mutation is to increase mTOR signaling.

While the consequences of increased ERK/PI3K/mTOR signaling are consistent with many of the anatomic and neuropathologic findings in ASD (e.g., excess brain growth and neuronal proliferation), it must be noted that this pathway is a central cellular regulator in most organ systems and pathogenic mutations in key members produce more diverse, severe, and widespread clinical manifestations than is generally seen in non-syndromic or idiopathic ASD. However, this convergence on a single molecular pathway is considered a significant clue to the pathogenesis of idiopathic ASD. It is likely that many ASD mutations occur in genes further upstream, thereby imparting a more subtle and brain region specific orientation to the downstream effect of mTOR activation. Probably the most studied and well known of such upstream activators is a receptor tyrosine kinase coded by the MET gene, located in the 7q31 ASD candidate region. MET is known to be important in forebrain development and exhibits altered expression in ASD cortical tissue (Campbell et al., 2007). A common promoter variant which affects MET function *in vitro* (Campbell et al., 2006), as well as a number of MET mutations, has been found to be associated with a subset of ASD cases (Campbell et al., 2009).

### ALTERATIONS IN SYNAPSE-RELATED GENES ARE ASSOCIATED WITH ASD

A second model for the pathogenesis of ASD focuses on abnormal formation or function of synaptic connections. This was first suggested by findings of abnormal dendritic spine morphology in the above syndromic forms of ASD as well as the high prevalence of seizures in both syndromic and idiopathic ASD. This model was supported by the identification of NLGN3, NLGN4X, NRXN1, and SHANK3 in ASD candidate loci. These are all synaptic cell adhesion molecules (CAMs) which are crucial for the dendrite development, initial contact between pre- and postsynaptic neurons, and/or assembly and anchoring of synaptic scaffolding proteins (reviewed by Betancur et al., 2009; Bourgeron, 2009). Overall, alterations in most candidate CAM genes do not appear to account for an appreciable proportion of ASD individually and are as likely to be found in association with other conditions or non-affected individuals alike. Additionally, single gene mouse models of these synaptic candidates usually have no discernable behavioral phenotype, although this alone does not exclude any candidate gene as potentially contributing to risk for ASD in humans.

Numerous other CAMs and synaptic scaffolding proteins are also under investigation as ASD susceptibility genes. These include various cadherins and protocadherins, members of the Ig CAM superfamily (e.g., L1CAM), and the contactins. One functional grouping (SHANK2/3, SYNGAP1, DLGAP2) converge on the postsynaptic density. Additionally, recent large-scale molecular and functional pathway analyses of CNV and association candidates (e.g., Pinto et al., 2010; Gilman et al., 2011; Hussman et al., 2011) have identified large functional groups converging on regulation of actin filament network dynamics. One group specifically, the Rho family of small GTPases, is particularly central to this process and therefore essential to dendrite morphogenesis and spine remodeling.

### ALTERATIONS IN GENES REGULATING NEURONAL POLARITY, NEURITIC OUTGROWTH, AND AXONAL

#### Guidance are associated with ASD

**FIGURE 2:**
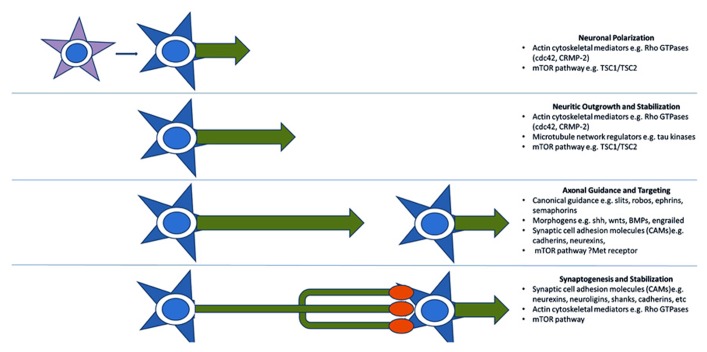
**Key molecular pathways of axonal development implicated by genetic studies.** Genome-wide association and CNV studies have implicated numerous molecules and molecular pathways involved in neuritic outgrowth, neuronal polarity, axonal-dendritic targeting, and synaptogenesis. Many of these are common to multiple related developmental processes as they serve the more general functions of providing positional information. This recycling phenomenon may explain the link between arealization/proliferation abnormalities, axonal and dendritic abnormalities, and synaptic dysfunction in ASD.

A third model for the pathogenesis of ASD, more recently advanced, reinterprets many of the above functional groupings in terms of axon outgrowth, guidance, and targeting. Many of these proteins can be thought of more generally as providing positional information and mediating motility and are, therefore, re-cycled for various developmental processes mechanistically requiring specific recognition and/or movement (**Figure 2**). An axonal model is therefore also supported by the identification of many of the aforementioned synaptic CAMs (e.g., L1CAM, SHANKs, and NRXN1), which are often involved in neuritic outgrowth and axon guidance and targeting (Sheng and Kim, 2000; Gjorlund et al., 2012; Tagliavacca et al., 2013). The Rho-GTPases and their regulators also act long before synaptogenesis to induce neurite formation and differentiation, mediate axonal extension and branching, and cause growth cone collapse in response to repulsive axonal guidance cues (Gilman et al., 2011). They do this by coordinating the interactions between the actin cytoskeleton of the axonal growth cone which interprets CAM-based guidance cues, and the microtubule network which stabilizes the growing neurite (Govek et al., 2011). Two recent ASD candidates, cdc42 and CRMP-2 (Gilman et al., 2011) are particularly important in early neuronal polarization, i.e., the differentiation of early neuritic processes into a single axon and multiple dendrites. This process forms the basis of directional information flow in neuronal circuits (Govek et al., 2011). Alterations in expression of these candidates in developing neurons causes either inhibition of axon formation or the production of supernumerary axons (Govek et al., 2011). No doubt as more “synaptic” molecules are investigated more closely in terms of developmental expression, axonal functions will continue to come to light.

The mTOR pathway, while important for synaptic function, it is also critical for neuritic growth and neuronal polarity. TSC1 and TSC2, mutated in tuberous sclerosis, form a complex which permits the functioning of a TSC2 GTPase activating protein for Rheb GTPase which inhibits the mTOR pathway. TSC pathway components are expressed in a polarized manner in developing neurons so that TSC2 is inactivated (and mTOR activity is increased) in the developing axon (Choi et al., 2008). Choi et al. (2008) found that overexpression of Tsc1/2 significantly inhibited axon formation in cultured mouse hippocampal neurons. Knockdown of Tsc2 and knockout of Tsc1 in hippocampal cultures, conversely, caused developing neurons to have multiple axons. This was born out *in vivo* by examinations of cortical sections derived from Tsc1^-^^/^^-^ mice, which have relatively a normal cortical and hippocampal architecture, but develop seizures at postnatal day 5 and die in a few weeks. Neurofilament stained sections demonstrated numerous ectopic axons throughout the cortex of these mice, even in the usually dendrite-rich upper layers (Choi et al., 2008).

The Met receptor, long known to be present (at low levels) and active in synapses of the mature brain (Tyndall and Walikonis, 2006) has now been found to be more highly expressed, before most synaptogenesis occurs, in extending forebrain axons of the developing mouse brain. Judson et al. (2009) demonstrated peak Met expression by Western blot at birth in the developing mouse brain; the period at which neurons are finished migrating and are actively extending axons and dendrites. These levels declined during synaptogenesis to low, adult baseline levels (Judson et al., 2009). Strong mRNA expression of Met was visualized in cortical neurons of layers II/III and V/VI and exhibited a strong caudal (high) to rostral (low) gradient in the cortical plate but uniform expression in the subplate. Protein expression by immunohistochemistry was visualized in the callosal, cingulated, anterior commissure, and internal and external capsule white matter tracts as well as in axons extending from the hippocampus, septum, and amygdala (Judson et al., 2009). No appreciable dendritic or synaptic staining was detected with this method.

Recently published association and CNV studies have also identified numerous candidate genes coding for canonical axonal guidance molecules including multiple members of the Slit (Duvall et al., 2006; Hu et al., 2009), Robo (Anitha et al., 2008), Ephrin (Sbacchi et al., 2010), and Semaphorin (Melin et al., 2006; Sbacchi et al., 2010) families. Sbacchi et al. (2010) used gene ontogeny and pathway analyses to determine common functions of duplicated or deleted genes lying within CNVs derived from four large ASD microarray data sets. They identified a substantial number of canonical axonal guidance genes as well as certain BMP, Wnt, Engrailed morphogens which are also known to participate in axon guidance (Charron and Tessier-Lavigne, 2005) and linked to ASD by previous studies (Kalkman, 2012). Hussman et al. (2011), similarly identified a substantial group of ASD candidate genes involved in neurite outgrowth by genome-wide association. Specific functions included axonal guidance, Rho-GTPase signaling, cytoskeletal regulation, and cadherin–catenin function. Interestingly, while different canonical axonal guidance genes are implicated in different studies, SEM5A appears to be listed in practically all of them. Sema5a has also been recently found to be enriched in the mouse subplate during development along with other ASD candidates such as Nrxn1, and cadherins 10, 18, and 9 (Hoerder-Suabedissen et al., 2013).

## CONCLUSION

Structural studies of brain development indicate a large subset of individuals with ASD experience dramatic overgrowth of frontal white matter in the first years of life. Excess fetal neuronal proliferation is likely responsible for much of the added volume, but may not explain abnormalities of white matter integrity and microstructure seen by DTI and microscopy. Abnormalities that persist into adult life, even as volumes “normalize.” Frontal and temporal cortical areas overlying this white matter are not as functionally integrated with more posterior cortical regions. Subtle (for the most part) abnormalities of cortical neuronal migration and lamination are variably seen, but there is little consistency in the findings. An exception to this is a relatively frequent excess of INs, presumed remnants of the fetal subplate. This excess may be a function of a general over-proliferation of cortical neurons or a reflection of aberrant axonal and/or synaptic connectivity during fetal life causing a subsequent failure of appropriate developmental apoptosis. Certainly, morphologic abnormalities reported in superficial subcortical white matter axons indicate a possible role for disordered organization of cortical afferent and/or efferent wiring through the subplate region.

Recently published association and CNV studies have identified, not only multiple axonal guidance molecules, but also numerous ASD candidate genes involved in neuritic outgrowth, neuronal polarity, and axonal–dendritic targeting. These include various participants in the mTOR signaling cascade, neuronal CAMs, Rho-GTPases, and traditional morphogens known to mediate axonal guidance. Many of these, particularly the CAMs and morphogens, can be thought of more generally as providing positional information, cues that may be variously interpreted by responding cells as division, fate specification, migration, neuritic sprouting/pathfinding, or synaptogenesis signals. In other words, they are re-cycled for various developmental processes mechanistically requiring positional information. Other candidates, such as those involved in mTOR and Rho-GTPase signaling, mediate neuronal interpretation of positional information and direct the response in a context-dependent manner. This recycling phenomenon may explain the link between arealization/proliferation abnormalities (frontal overgrowth), axonal and dendritic abnormalities, and synaptic dysfunction in ASD. Current interpretations of the genetic and neuropathologic data are more a matter of emphasis than mutual exclusion, however, the concept of a significant axonal component to the pathogenesis of ASD should be considered in constructing a model that encompasses all of the clinical, structural, and functional observations.

## Conflict of Interest Statement

The authors declare that the research was conducted in the absence of any commercial or financial relationships that could be construed as a potential conflict of interest.
